# Long‐term outcomes in Japanese nonagenarians undergoing transcatheter aortic valve implantation: A multi‐center analysis

**DOI:** 10.1002/clc.23183

**Published:** 2019-04-23

**Authors:** Hiroaki Yokoyama, Tetsuya Tobaru, Yuki Muto, Kenichi Hagiya, Ryosuke Higuchi, Mike Saji, Itaru Takamisawa, Jun Shimizu, Shuichiro Takanashi, Morimasa Takayama, Hirofumi Tomita, Harutoshi Tamura, Shinichiro Doi, Shinya Okazaki, Mitsuaki Isobe

**Affiliations:** ^1^ Department of Cardiology Sakakibara Heart Institute Tokyo Japan; ^2^ Department of Cardiology Hirosaki University Graduate School of Medicine Hirosaki Japan; ^3^ Department of Cardiology Kawasaki Saiwai Hospital Kawasaki Japan; ^4^ Department of Anesthesiology Sakakibara Heart Institute Tokyo Japan; ^5^ Department of Cardiovascular Surgery Sakakibara Heart Institute Tokyo Japan; ^6^ Department of Cardiology, Pulmonology, and Nephrology Yamagata University School of Medicine Yamagata Japan; ^7^ Department of Cardiovascular Medicine Juntendo University School of Medicine Tokyo Japan

**Keywords:** long‐term outcome, nonagenarians, TAVI

## Abstract

**Background and Hypothesis:**

Japan is an aging society, and the number of nonagenarians with severe aortic stenosis undergoing transcatheter aortic valve implantation (TAVI) is increasing, but their outcomes have not been determined fully.

**Methods:**

We prospectively enrolled 767 consecutive patients who underwent TAVI in three Japanese institutions. Clinical characteristics and outcomes of nonagenarians (n = 94) were evaluated and compared with those of patients aged <90 years (n = 673).

**Results:**

Prevalence of New York Heart Association (NYHA) class III/IV was not different between the two groups. Preoperative risk scores were significantly higher in nonagenarians compared with those in non‐nonagenarians, whereas the Clinical Frailty Scale was not different. Thirty‐day mortality tended to be higher (*P* = .06) and major vascular complication was significantly higher in nonagenarians than in non‐nonagenarians (*P* < .05), but 3‐year mortality was equivalent between the two groups. Even after adjustment for covariates, female sex (hazard ratio, 0.41; 95% confidence interval: 0.25‐0.67), body mass index (0.87, 0.80‐0.94), and NYHA class III/IV (1.84, 1.06‐3.29) were associated with all‐cause mortality. Age ≥ 90 years was not associated with all‐cause mortality.

**Conclusions:**

TAVI could be undertaken safely and effectively in nonagenarians, who had acceptable long‐term results compared with those for younger patients, although careful attention should be paid to major vascular complication.

## 1 INTRODUCTION

The number of older patients with aortic stenosis (AS) is increasing. Their outcomes, with symptoms such as angina pectoris, syncope, or congestive heart failure, are very poor unless surgical aortic valve replacement (SAVR) is undertaken.^1^ Increasingly, in developed countries, older patients are presenting with multiple comorbidities, making them high‐risk surgical candidates. The perioperative mortality of SAVR increases with age up to about 10% in patients aged ≥ 90 years.^2^

Transcatheter aortic valve implantation (TAVI) has emerged as a less invasive alternative to SAVR in patients with severe AS who cannot undergo surgery, or who are at intermediate/high surgical risk, resulting in acceptable clinical outcomes.^3^, ^4^, ^5^, ^6^, ^7^, ^8^ Japan is an aging society, and the number of nonagenarians with severe AS undergoing TAVI is increasing, but their outcomes have not been determined fully.

Vendrik et al^9^ reported the 5‐year outcomes of European nonagenarians who underwent TAVI. Moreover, Miura et al^10^ reported on the early outcomes of Japanese nonagenarians who underwent TAVI, but they did not evaluate long‐term outcomes. In this study, we evaluated the long‐term outcomes of Japanese nonagenarians who underwent TAVI.

## 2 METHODS

### 2.1 Study patients

We prospectively enrolled 767 consecutive patients with severe symptomatic AS who presented to Sakakibara Herat Institute (n = 625), Yamagata University Hospital (n = 42), or Juntendo University Hospital (n = 100), all of which are located in Japan, between April 2010 and September 2018 and underwent TAVI. This study protocol was conducted according to the principles of the Declaration of Helsinki and approved by the ethics committees of each of these three institutions.

The decision to undertake TAVI was made by the Cardiac Teams (cardiologists, cardiac surgeons, radiologists, and anesthesiologists) of each institution. All patients were considered to be “high‐risk” cases and not suitable for SAVR by the Cardiac Teams. The Society of Thoracic Surgeons predictive risk of mortality, the logistic European System for Cardiac Operative Risk Evaluation (Logistic EuroScore), and Euro II Score were evaluated for preoperative risk. Moreover, the Clinical Frailty Scale (CFS), major organ dysfunction (including the respiratory system) and procedure‐specific impediments were evaluated to confirm patient status not reflected in preoperative risk scores according to the 2014 American Heart Association/American College of Cardiology guidelines for the management of valvular heart disease.^11^

The transfemoral approach was the primary procedure. Selection and sizing of the device were based on multi‐slice computed tomography by each Cardiac Team. Other access sites (eg, transapical or trans‐subclavian artery) were considered if the transfemoral approach was not suitable for advancing the large sheath.

### 2.2 Endpoints

The primary endpoint of our study was all‐cause mortality after TAVI. Moreover, we evaluated early safety endpoints (all‐cause mortality, stroke, life‐threatening bleeding, acute kidney injury, coronary artery obstruction, major vascular complications, and valve‐related dysfunction requiring repeat procedure) according to Valve Academic Research Consortium 2 (VARC‐2) criteria.^12^ Information regarding patient survival was obtained from each institution where TAVI was done or through telephone calls directly to patients/patients' families according to the criteria of the ethics committee of each participating institution.

### 2.3 Statistical analyses

Continuous variables are the mean ± SD or median (interquartile range). Categorical variables are expressed as numbers and percentages. An unpaired *t* test or *χ* ^2^ test was used to compare differences between the two groups. The Mann‐Whitney *U* test was used for nonparametric variables. The chi‐square test was used to compare categorical variables. Mortality was estimated using the Kaplan‐Meier method and compared using the log‐rank test. Multivariate analysis for the predictors of all‐cause mortality 3 years after TAVI was undertaken using Cox proportional hazards regression. The variables used for this analysis were age ≥ 90 years, female sex, body mass index (BMI), New York Heart Association (NYHA) class III/IV, CFS ≥ 4, EuroScore II, estimated glomerular filtration rate (eGFR), left ventricular ejection fraction (LVEF), and presence of chronic obstructive pulmonary disease. Hazard ratios (HRs) and 95% confidence intervals (CIs) were calculated. Statistical analyses were carried out using JMP 13 (SAS, Cary, NC, USA). *P* < .05 was considered significant.

## 3 RESULTS

### 3.1 Baseline characteristics of the study cohort

We evaluated 767 patients (543 females [71%]; mean age, 84 ± 5 years) and they were divided into two groups according to their age at TAVI: nonagenarians (age ≥ 90 years, n = 94) and non‐nonagenarians (age < 90 years, n = 673). The baseline characteristics of study patients are summarized in Table 1. There was no significant difference in sex between the two groups. Nonagenarians were shorter and lighter, and had a lower BMI than non‐nonagenarians. A history of stroke, coronary artery disease, peripheral arterial disease, and atrial arrhythmia were not significantly different between the two groups. Prevalence of NYHA class III/IV was not significantly different between the two groups. EuroScore II (5.5 [3.8‐7.1] vs 3.9 [2.5‐6.5], Logistic EuroScore (17.0 [14.4‐25.1] vs 12.1 [9.0‐18.5]), and STS PROM (8.6 [6.8‐11.7] vs 5.5 [3.8‐7.6]) were significantly higher in nonagenarians compared with those in non‐nonagenarians. The CFS was not significantly different between the two groups.

**Table 1:** Baseline characteristics of the study cohort

Variables	Overall (n = 767)	Age ≥ 90 (n = 94)	Age < 90 (n = 673)	*P* value
Age, year	84 ± 5	92 ± 2	83 ± 5	<.05
Female gender n, (%)	543 (71)	68 (72)	475 (71)	.81
Height (cm)	150.4 ± 9.1	147.7 ± 8.5	150.8 ± 9.1	<.05
Weight (kg)	50.4 ± 9.9	46.0 ± 8.0	51.1 ± 10.0	<.05
BMI, kg/m^2^	22.2 ± 3.7	21.0 ± 2.8	22.4 ± 3.7	<.05
Medical history				
Hypertension, n (%)	594 (77)	68 (72)	526 (78)	.24
Dyslipidemia, n (%)	420 (55)	43 (46)	377 (56)	.08
Diabetes mellitus, n (%)	177 (23)	15 (16)	162 (24)	.09
NYHA class III/IV, n (%)	409 (53)	54 (57)	355 (53)	.44
Previous stroke, n (%)	83 (11)	6 (6)	77 (11)	.16
Previous myocardial infarction, n (%)	44 (6)	3 (3)	41 (6)	.35
Previous coronary intervention, n (%)	139 (18)	18 (19)	121 (18)	.78
Previous bypass surgery, n (%)	49 (6)	2 (2)	47 (7)	.07
COPD, n (%)	55 (7)	9 (10)	46 (7)	.39
Atrial fibrillation/flutter, n (%)	180 (23)	27 (29)	153 (23)	.20
Peripheral vascular disease, n (%)	119 (16)	14 (15)	105 (16)	1.0
Permanent pacemaker, n (%)	45 (6)	7 (7)	38 (6)	.48
Calculated risk scores				
EuroScore II, %	4.2 [2.6‐6.7]	5.5 [3.8–7.1]	3.9 [2.5–6.5]	<.05
Logistic EuroScore, %	12.8 [9.5‐19.0]	17.0 [14.4–25.1]	12.1 [9.0–18.5]	<.05
STS‐PROM, %	5.8 [4.0‐8.3]	8.6 [6.8‐11.7]	5.5 [3.8‐7.6]	<.05
Clinical frailty scale ≥4, n (%)	554 (72)	70 (74)	484 (72)	.71
Echo parameters (Pre‐TAVI)				
Aortic valve area, cm^2^	0.65 ± 0.16	0.60 ± 0.17	0.65 ± 0.16	<.05
Aortic valve max gradient, mm Hg	92.4 ± 31.4	96.7 ± 34.3	91.8 ± 30.9	.16
Aortic valve mean gradient, mm Hg	53.1 ± 19.2	55.5 ± 20.9	52.8 ± 18.9	.20
LVEF, %	60.3 ± 9.9	59.4 ± 9.6	60.4 ± 9.9	.36
Aortic valve regurgitation grade	1.4 ± 0.7	1.3 ± 0.7	1.4 ± 0.7	.52
Laboratory data				
eGFR, mL/min/1.73m^2^	54.3 ± 18.9	48.4 ± 16.3	55.1 ± 19.1	<.05
Hemoglobin, g/dL	11.6 ± 1.5	11.4 ± 1.2	11.6 ± 1.6	.23
Albumin, g/dL	3.8 ± 0.4	3.7 ± 0.4	3.8 ± 0.4	.39
NT‐proBNP, pg/mL	1259 [534‐3276]	2200 [911‐4942]	1154 [515‐3017]	<.05

*Note*. Values are the mean ± SD or the median [IQR].

Abbreviations: BMI, body mass index; COPD, chronic obstructive pulmonary disease; eGFR, estimated glomerular filtration rate; LVEF, left ventricular ejection fraction; NT‐proBNP, N‐terminal prohormone of brain natriuretic peptide; NYHA, New York Heart Association; STS‐PROM, Society of Thoracic Surgeons predictive risk of mortality; TAVI, transcatheter aortic valve implantation.

We also assessed transthoracic echo parameters before TAVI. The area of the aortic valve was smaller in nonagenarians compared with non‐nonagenarians (0.60 ± 0.17 vs 0.65 ± 0.16 cm^2^, *P* < .05), but there was no significant difference in LVEF or the prevalence of aortic valve regurgitation. The level of N‐terminal prohormone of brain natriuretic peptide (NT‐proBNP) was higher, and eGFR lower, in nonagenarians than in non‐nonagenarians.

### 3.2 TAVI

The procedural characteristics of TAVI are summarized in Table 2. A transfemoral approach was taken in 678 (88%) patients, and there were no significant differences in approach between the two groups. In the patients with transfemoral approach, 100 patients (15%) were treated with cutdown approach. Moreover, about half of all patients were treated with general anesthesia, and there were no significant differences in anesthesia methods between the two groups. Of 767 patients, 745 (97%) achieved device success, and device success was lower in nonagenarians than in non‐ nonagenarians (94% vs 98%, *P* < .05). In nonagenarians, six patients (6%) did not achieve device success: one patient died due to annulus rupture; three patients did not achieve correct positioning of a single prosthetic heart valve into the appropriate anatomic location; two patients did not achieve the intended performance of the prosthetic heart valve. In patients aged <90 years, 16 patients (2%) did not achieve device success: one patient died due to acute limb ischemia; 10 patients did not achieve correct positioning of a single prosthetic heart valve into the appropriate anatomic location; five patients did not achieve the intended performance of the prosthetic heart valve.

**Table 2:** TAVI characteristics

Variables	Overall (n = 767)	Age ≥ 90 (n = 94)	Age < 90 (n = 673)	*P* value
Transcatheter heart valve, n (%)				<.05
Sapien XT	214 (28)	28 (30)	186 (28)	
Lotus	11 (1)	4 (4)	7 (1)	
CoreValve	29 (3)	3 (3)	26 (4)	
S3	363 (47)	40 (43)	323 (48)	
Evolute R	136 (18)	13 (14)	123 (18)	
Acurate	1 (1)	0 (0)	1 (1)	
Evolute PRO	11 (1)	5 (5)	6 (1)	
Approach site, n (%)				.32
Transfemoral	678 (88)	85 (90)	593 (88)	
Transapical	74 (10)	6 (6)	68 (10)	
Others	15 (2)	3 (3)	12 (2)	
Anesthesia, n (%)				.44
General	405 (53)	46 (49)	359 (53)	
Local	362 (47)	48 (51)	314 (47)	
Device success	745 (97)	88 (94)	657 (98)	<.05

*Note*. “Device success” was defined as the composite endpoint according to VARC‐2 criteria.

### 3.3 Early outcomes

**Figure 1:**
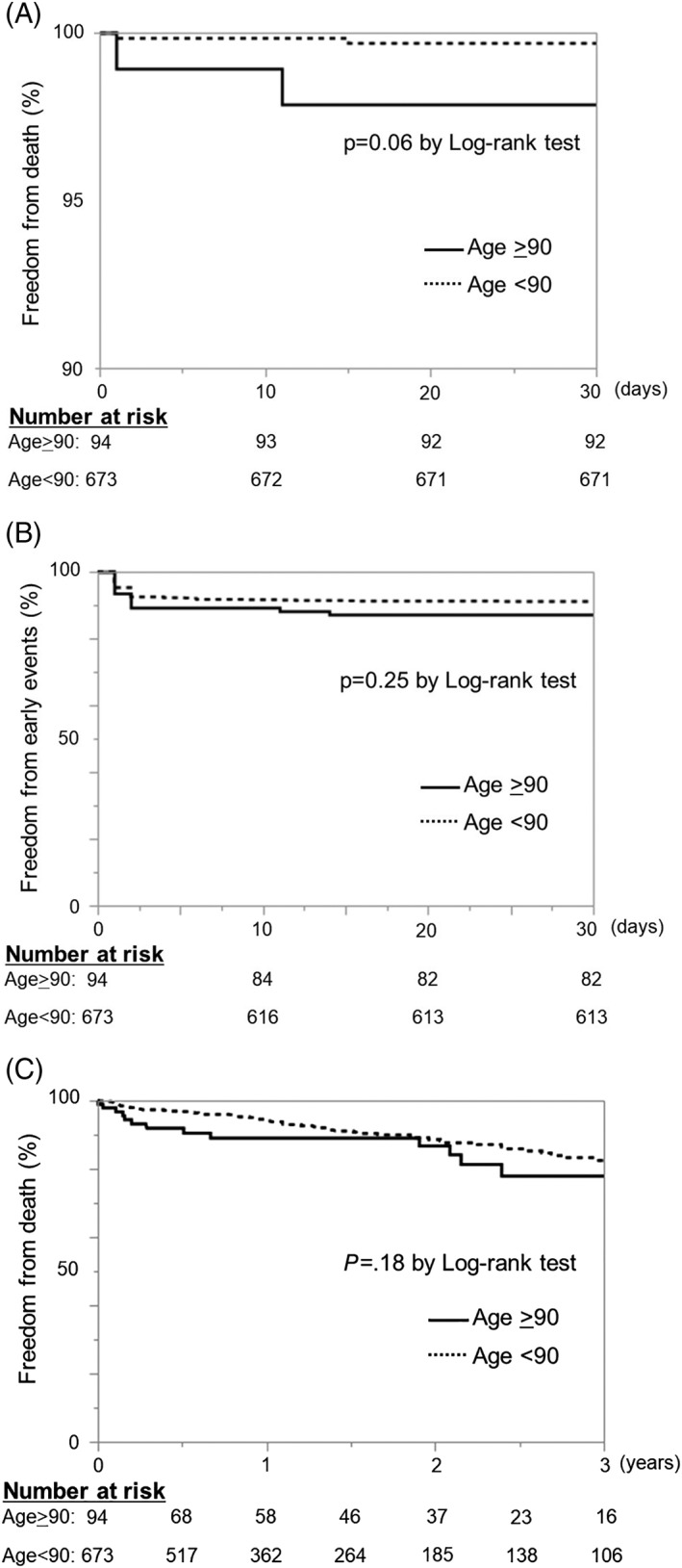
Kaplan‐Meier curves of: A, All‐cause mortality at 30 days stratified by the age at procedure; B, Early safety endpoints stratified by the age at procedures, and C, All‐cause mortality at 3 years stratified by the age at procedure

Early outcomes are summarized in Table 3. Five patients died of any cause 30 days after TAVI. Survival at 30 days tended to be lower in nonagenarians compared with non‐nonagenarians but not significantly so (97.9% in nonagenarians vs 99.6% in non‐nonagenarians; log‐rank test, *P* = .06) (Figure 1A). Early safety according to VARC‐2 criteria was 10%, and there was no significant difference between the two groups (Figure 1B). The prevalence of stroke, life‐threatening bleeding, acute kidney injury (stage 2 or 3), coronary artery obstruction or valve‐related dysfunction was similar in both groups. A major vascular complication was significantly more prevalent in nonagenarians than in non‐nonagenarians (19% vs 1%, *P* < .05). The duration of hospital stay after TAVI was similar in both groups (10 [7–18] in nonagenarians vs 9 [7–14] days in non‐nonagenarians).

**Table 3:** Early outcomes and long‐term mortality of TAVI

Variables	Overall (n = 767)	Age ≥ 90 (n = 94)	Age < 90 (n = 673)	*P* value
30‐day outcome, n (%)				
All‐cause mortality	5 (1)	2 (2)	3 (1)	.06
Stroke	28 (4)	6 (7)	22 (3)	.13
Life‐threatening bleeding	19 (3)	4 (4)	15 (2)	.23
Acute kidney injury‐stage 2 or 3	12 (2)	2 (2)	10 (2)	.62
Coronary artery obstruction	6 (1)	1 (1)	5 (1)	.73
Major vascular complications	25 (3)	18 (19)	7 (1)	<.05
Valve‐related dysfunction	2 (1)	1 (1)	1 (1)	.10
Early safety, n (%)	73 (10)	12 (13)	61 (9)	.25
Hospital stay after procedure, days	9 [7‐15]	10 [7–18]	9 [7–14]	.14
Long‐term mortality				
All‐cause mortality	71 (18)	13 (22)	58 (18)	.19
Cardiovascular death	27 (4)	8 (9)	19 (3)	
Non‐cardiovascular death	41 (5)	5 (5)	36 (5)	
Unknown	3 (1)	0 (0)	3 (1)	

*Note*. “Early safety” was defined as the composite endpoint according to VARC‐2 criteria.

### 3.4 Long‐term mortality

Long‐term mortality was evaluated by the log‐rank test (Figure 1C). Freedom from any cause of death 3 years after TAVI was not significantly different between the two groups (77.9% in nonagenarians vs 82.5% in non‐nonagenarians, *P* = .25), and the cause of death (cardiovascular death or non‐cardiovascular death) was mentioned in Table 3.

### 3.5 Predictors of all‐cause mortality

We performed multivariate Cox proportional hazard analyses to evaluate the predictors of all‐cause mortality 3 years after TAVI (Table 4). Even after adjustment for covariates, female sex (HR, 0.41; 95%CI: 0.25‐0.67), BMI (0.87, 0.80‐0.94), NYHA class III/IV (1.84, 1.06‐3.29), and eGFR (0.98, 0.97‐0.99) were independently associated with all‐cause mortality. Age ≥ 90 years was not associated with all‐cause mortality.

**Table 4:** Adjusted hazard ratios for all‐cause mortality 3 years after TAVI

	Adjusted HR (95% CI)	*P* value
Age ≥ 90 years	1.23 (0.64‐2.20)	.51
Female sex	0.41 (0.25–0.67)	<.05
BMI	0.87 (0.80‐0.94)	<.05
NYHA class III/IV	1.84 (1.06–3.29)	<.05
Clinical frailty scale ≥4	1.03 (0.60‐1.86)	.92
EuroScore II	1.03 (0.99‐1.05)	.11
COPD	1.54 (0.70‐3.02)	.27
eGFR	0.98 (0.97–0.99)	<.05
LVEF	1.01 (0.99‐1.04)	.39

Abbreviations: BMI, body mass index; CI, confidence interval; COPD, chronic obstructive pulmonary disease; eGFR, estimated glomerular filtration rate; HR, hazard ratio; LVEF, left ventricular ejection fraction; NYHA, New York Heart Association, TAVI; transcatheter aortic valve implantation.

## 4 DISCUSSION

In this three‐center study, we evaluated the long‐term outcomes of Japanese nonagenarians who underwent TAVI. Main findings were that: (a) patients aged ≥90 years tended to have higher 30‐day mortality in comparison with those with aged <90 years; (b) early safety was similar between patients aged ≥90 years and those aged <90 years; (c) there was no significant difference in 3‐year outcomes between patients aged ≥90 years and those aged <90 years; (d) age ≥ 90 years was not a predictor for all‐cause mortality. Nonagenarians had a slightly higher prevalence of short‐term mortality, but there was not significantly different in long‐term mortality between nonagenarians and non‐nonagenarians. Hence, caution in selecting TAVI for nonagenarians might be unwarranted.

There were few differences in preoperative comorbidities as evaluated by the CFS between nonagenarians and non‐nonagenarians. Considering these results, it appears that the nonagenarians in this study are self‐selected patient populations who have selecting bias. Age is an important prognostic factor and should be taken into consideration, but comorbidities or functional status have been shown to be better predictors of mortality.^13^ We think that careful evaluation of patients and their risk factors before TAVI is very important for preventing postoperative complications, morbidity and mortality.

Several researchers have reported worse short‐term mortality in nonagenarians who underwent TAVI compared with that in non‐nonagenarians, but acceptable long‐term outcomes have been reported, especially for patients who underwent TAVI using a transfemoral approach.^9^, ^14^, ^15^, ^16^, ^17^, ^18^ Those results support our data showing that patients aged ≥90 years tended to have higher 30‐day mortality in comparison with those aged <90 years, but there was no significant difference in 3‐year outcome in our study.

TAVI‐based complications and postoperative morbidity might affect the quality of life of nonagenarians who undergo TAVI. Vascular problems are common and frequent complications of TAVI. Similar to previous studies, we found that major vascular complications were more frequent in nonagenarians than in patients aged <90 years.^14^, ^19^ The prevalence of life‐threatening bleeding was similar in both groups, and the proportion of patients who needed transfusion for any reason was not significantly different between patients aged ≥90 (21%) and those aged <90 years (24%). In contrast, Havakuk et al^20^ reported a similar risk of major vascular complications in patients aged >85 years and those aged <85 years. Reducing the risk of vascular complications for nonagenarians who undergo TAVI is an important issue that merits further investigation.

Stroke is a major complication and results in worse morbidity and mortality of patients who undergo TAVI. In the present study, stroke prevalence in hospital or within 30 days was 4% in all patients, and there was no significant difference between nonagenarians and non‐nonagenarians. These results are similar to those from other researchers.^14^, ^16^, ^17^ Vendrik et al^9^ reported that nonagenarians had more postoperative strokes (<72 hours) compared with younger counterparts. Our results regarding a similar prevalence of post‐TAVI stroke between nonagenarians and non‐nonagenarians might reflect an equivalent hospital stay after TAVI.

Langanay et al^21^ reported that the percentage of nonagenarians who underwent SAVR was only 1% of the total population. In the present study, the percentage of nonagenarians who underwent TAVI was 12%, quite different from the age distribution of patients with SAVR. The decision to carry out SAVR might depend on patients' age, so many patients aged ≥90 years received the benefit of TAVI. Moreover, nonagenarians who underwent TAVI had acceptable long‐term results compared with those aged <90 years, and multivariate analysis showed that age ≥ 90 years was not a predictor of long‐term mortality. We believe that hesitation in assigning nonagenarians to TAVI might be unwarranted, though consensus on evaluation of adaptation to TAVI by Cardiac Teams is needed.

In the present study, about half of all patients were treated by local anesthesia with mild sedation and without intubation. Benefits of local anesthesia include early recovery and risk reduction of hemodynamic instability during TAVI. Local anesthesia with mild sedation is favored, especially in nonagenarians, because they need a shorter stay in hospital to recover early after TAVI. It is thought that local anesthesia with mild sedation will aid better outcomes for nonagenarians who undergo TAVI.

Our study had two main limitations. First, there was selection bias among nonagenarians. No data were available among nonagenarians who did not undergo TAVI. Second, our study cohort was small, so further large‐scale studies are clearly warranted.

## 5 CONCLUSIONS

TAVI could be carried out safely and effectively in nonagenarians, and they had acceptable long‐term results compared with non‐nonagenarians, although careful attention should be paid to major vascular complication.

## CONFLICT OF INTEREST

The authors declare no potential conflict of interests.
